# Trends in attractiveness of general practice as a career: surveys of views of UK-trained doctors

**DOI:** 10.3399/bjgp17X689893

**Published:** 2017-03-14

**Authors:** Trevor W Lambert, Fay Smith, Michael J Goldacre

**Affiliations:** UK Medical Careers Research Group, Unit of Health-Care Epidemiology, Nuffield Department of Population Health, University of Oxford, Oxford.; UK Medical Careers Research Group, Unit of Health-Care Epidemiology, Nuffield Department of Population Health, University of Oxford, Oxford.; UK Medical Careers Research Group, Unit of Health-Care Epidemiology, Nuffield Department of Population Health, University of Oxford, Oxford.

**Keywords:** career choice, general practice, health workforce, hospital practice

## Abstract

**Background:**

It is current UK policy to expand the numbers of newly qualified doctors entering training to become GPs, to meet increased demand.

**Aim:**

To report on trends in young doctors’ views on the attractiveness of general practice as a career, compared with hospital practice.

**Design and setting:**

Questionnaire surveys in the UK.

**Method:**

Surveys of doctors, 3 years after graduation, conducted in successive year-of-qualification cohorts between 1999 and 2015.

**Results:**

The overall response rate from contactable doctors was 55%. In response to the statement *‘General practice is more attractive than hospital practice for doctors at present’*, 59% of doctors agreed in the 1999 survey, 77% in 2005, and only 36% in 2015. One-third of doctors agreed that their exposure to general practice had been insufficient for them to assess it as a career option, but this improved over time: agreement fell from 39% in 1999 to 28% in 2015. As a factor influencing specialty choice, enthusiasm for, and commitment to, the specialty was rated as very important by 65% of intending GPs in 2015, up from 49% in 1999; the corresponding figures for intending hospital doctors were 91% in 2015, up from 61% in 1999.

**Conclusion:**

Over the 16 years covered by this study, the attractiveness of general practice has fallen relative to hospital practice. This may not necessarily reflect a decline in attractiveness of general practice in absolute terms; rather, it may reflect a greater increase, over time, in the appeal of hospital practice.

## INTRODUCTION

The UK healthcare system requires that about 50% of new UK-trained graduates in medicine should enter family medicine as GPs.^1^ The percentages of recent medical graduates entering general practice training, however, have been much lower.^2^ General practice has been the first choice of future career specialty of only 20% of medical graduates, and this percentage has been largely unchanged over recent years.^3^

There are also retention problems with the GP workforce, with increasing numbers of GPs leaving.^2^ Older GPs, in particular, cite high workload, concerns about personal health, domestic factors, and organisational change including the requirement for revalidation as reasons for leaving direct patient care as a GP.^4^ Younger GPs account for 46% of leavers: those who leave often report an increasing administrative burden, a rising overall workload, and reduced consultation time with each individual patient as reasons for their decision.^5^

Primary care in the UK, in common with other high-income countries, faces the challenges of increased demand and workload because of a growing and ageing population, a rise at all ages in conditions that may be lifestyle related, and an expansion of the range of possible treatments.^6^^,^^7^ Against this backdrop, a ‘new deal for general practice’ was recently drawn up that aims to improve the recruitment, retention, and return to practice of GPs.^8^

Most recently, in April 2016, NHS England published its *General Practice Forward View*, which targeted a large increase in GP training posts and a major recruitment campaign in England to attract doctors to general practice.^9^

This study examines the views of doctors, 3 years after qualification, whether or not they chose a career in general practice, on the relative attractiveness of careers in general practice and in the hospital specialties. The same questions were posed to several successive year-of-graduation cohorts.

The aim of this study was to report on temporal trends in the attractiveness of general practice as a career, as reported by young doctors making their choice of future specialty career. Views of doctors who chose general practice were compared with views of those who chose other specialties, and views of male and female doctors were compared.

## METHOD

The UK Medical Careers Research Group undertook multipurpose surveys of the career intentions and views of all UK medical graduates of 1996, 2002, 2008, and 2012. Doctors were surveyed 3 years after graduation, in surveys which took place between 1999 and 2015. Up to four reminders were sent to non-responders. Methodological details are available elsewhere.^10^

In each survey doctors were asked to state their level of agreement with the following statement: *‘General practice is more attractive than hospital practice for doctors at present.’* Responses were collected on a 5-point scale covering *‘strongly agree’*, *‘agree’*, *‘neither agree nor disagree’, ‘disagree’*, and *‘strongly disagree’*, with a *‘no opinion’* option.

**How this fits in**

The percentages of new doctors entering general practice training are much lower than the 50% required by the UK healthcare system. There are also retention problems with the GP workforce. This study considered the views of doctors, 3 years after qualification, on whether or not they chose a career in general practice. It was found that the attractiveness of a career in general practice relative to hospital practice has declined substantially in recent years.

To enable the use of χ^2^ analysis by avoiding small cell counts, and to simplify the tabulation of data, this scale was reduced to a 3-point scale (with ‘*strongly agree*’ and ‘*agree*’ combined, and ‘*strongly disagree*’ combined with ‘*disagree*’). Two further statements were presented using the same 5-point scale of agreement: *‘My exposure to general practice has been insufficient for me to assess it as a career option’* (not presented to the graduates of 2008); and *‘Generally speaking, GP training in the UK is of a higher quality than training in the hospital specialties’* (only the 2012 graduates).

Doctors were asked to specify their choice of specialty for their eventual career. Doctors could nominate up to three choices and could indicate an order of priority and whether any of their choices were tied; that is, of equal priority. The present study focuses on first (untied) choices. Responders were grouped for analysis into four groups according to their first choice of career specialty: general practice, the hospital medical specialties, surgical specialties (including obstetrics and gynaecology), and other hospital-based specialties combined (paediatrics, emergency medicine, anaesthetics, radiology, clinical oncology, pathology, and psychiatry).

Each doctor rated the influence of each of 12 factors on their career choice (Box 1). The doctors were asked to indicate whether each factor had influenced their choice of specialty *‘not at all’*, *‘a little’*, or *‘a great deal’*.

Box 1. **Twelve factors affecting career choice**

**Table:** 

Wanting a career that fits my domestic situation Wanting a career with acceptable hours/working conditions Eventual financial prospects Promotion/career prospects Self-appraisal of own skills/aptitudes Advice from others Experience of chosen subject as student Inclinations before medical school Experience of jobs so far Enthusiasm/commitment: what I really want to do Availability of training places Availability of career posts

All factors were included in each of the four surveys except the last two, which were excluded from the survey of 2002 graduates, and ‘Promotion/career prospects’, which was excluded from the survey of the graduates of 2008.

Variation in agreement or disagreement about the attractiveness of general practice careers was examined by qualification cohort, sex, and specialty chosen. The data were analysed by univariate cross-tabulation. To test statistical significance χ^2^ statistics were used (reporting Yates’s continuity correction where appropriate).

## RESULTS

### Response rates

Surveys were sent to 20 527 UK doctors. The aggregated response rate from contactable doctors, over all four surveys, was 55% (10 766/19 501). Non-contactable included those with no address, deceased, not registered, or who had declined to take part in earlier surveys. Response to the four surveys fell in successive survey years and was, respectively, 72% (2721/3776), 65% (2748/4239), 49% (3228/6540), and 42% (2069/4946). Of 10 766 doctors who responded, 577 completed a short questionnaire that did not include any questions about general practice. These are excluded from the analysis.

The rest of this study focuses on the remaining 10 189 responders. Analysis by specialty is restricted to the 9161 doctors who made an untied first choice for either general practice or hospital practice.

### Responses to the statement ‘General practice is more attractive than hospital practice for doctors at present’

Overall, most doctors (60%) agreed or strongly agreed with the statement, 17% neither agreed nor disagreed, and 23% disagreed or strongly disagreed. There was a huge fall over time, however, in levels of agreement that general practice was more attractive than hospital practice, from 77% in 2005 to 36% in 2015 (Table 1), a pattern observed in both males and females.

**Table 1.** Percentages of responding doctors who agreed or strongly agreed with the statement *‘General practice is more attractive than hospital practice for doctors at present’* presented 3 years after graduation^a^

**Responders’ career choice**	**Sex**	**Year of survey**				

		**1999**	**2005**	**2011**	**2015**	**All years**
All specialties	Male and female	58.5	76.5	64.1	36.1	60.1
	Male	54.8	75.1	65.7	32.6	58.0
	Female	61.5	77.2	63.2	38.4	61.4

General practice	Male and female	89.0	92.6	81.4	51.1	79.7
	Male	86.3	93.0	82.7	47.1	77.3
	Female	90.3	92.4	80.9	53.1	80.7

Hospital practice	Male and female	44.7	69.8	55.5	28.9	50.9
	Male	45.6	71.3	59.6	27.0	52.0
	Female	43.9	68.7	52.7	30.3	50.1

a *Based on responses from 9776 doctors (all specialties), 2793 (general practice), and 6016 (hospital practice). Responders in general practice or hospital practice in each survey year. Excludes 82 who specified ‘no opinion’ and 331 who did not answer the statement. Responders in each survey year: 1999 1054 male, 1248 female, total 2302; 2005 723 male, 1283 female, total 2006; 2011 1039 male, 1760 female, total 2799; 2015 672 male, 1030 female, total 1702. Appendix 1 shows the numbers and percentages in each response category. Statistical tests on the percentages strongly agreeing or agreeing: 1) male versus female, all years combined: all career choices χ^2^ (1 degree of freedom [df])* = *12.4,* P = *0.0004; general practice choices χ^2^ (1 df) = 3.9,* P = *0.047; hospital practice choices χ^2^ (1 df) = 1.9,* P = *0.17; 2) year of survey comparisons on each row of the table (χ^2^ (3 df) tests): all* P*<0.001.*

Doctors whose specialty choice was general practice were much more likely to agree with the statement (80%) than doctors who chose a hospital specialty (51%), but in both specialty choice groups there was a substantial decline in agreement between 2005 and 2015 (Table 1). Among doctors who chose general practice, only 51% of doctors surveyed in 2015 agreed that general practice was more attractive than hospital practice, compared with 93% of doctors surveyed in 2005. Among doctors choosing hospital practice, the corresponding fall was from 70% in 2005 to 29% in 2015. Within each career choice group and survey year the difference by sex was small (see footnotes to Table 1 for test results and Appendix 1 for details of all five response categories).

### Factors influencing specialty choice

**Figure 1.**
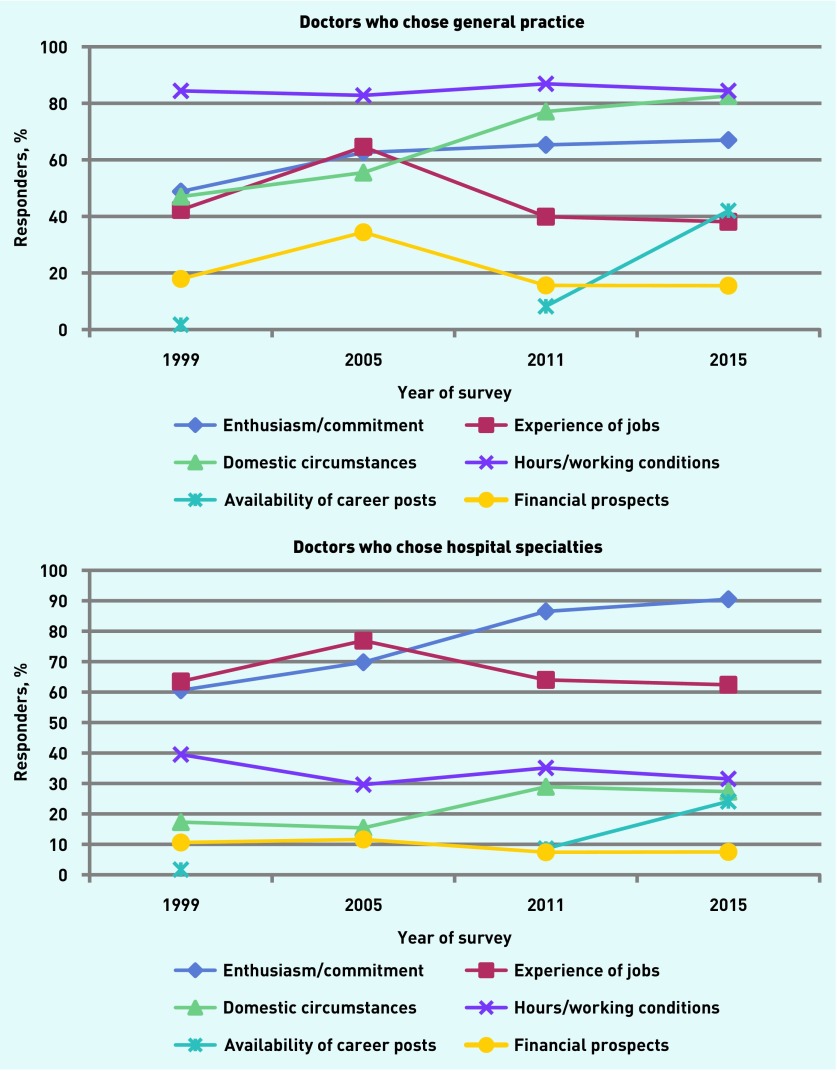
***Trends between 1999 and 2015 in factors affecting career choices, for doctors who chose general practice and hospital practice: percentages of responders in each survey cohort who stated that each factor had a great deal of influence on their career choice. Availability of career posts was excluded from the survey of the 2002 graduates.***

The percentages were compared in each survey of those who stated that each of 12 factors had affected their career choice *‘a great deal’* (Figure 1 and Appendix 2), with specific interest in absolute levels of influence, differences between aspiring GPs and aspiring hospital doctors, and trends over time. Enthusiasm for, and commitment to, the specialty ranked highly both for GPs and hospital specialties, with higher scores for the latter than the former. This was scored as influencing choice a great deal by 67% of GPs in 2015 (up from 49% in 1999 to 63% in 2005, but with no appreciable change since then). By contrast, enthusiasm for their chosen hospital specialty was rated very highly by 91% of hospital doctors (up from 61% in 1999, 70% in 2005, 87% in 2011). Another factor that hospital doctors rated much more highly than GPs was *‘experience of jobs so far’*: in 2015 this was *‘a great deal’* of importance for 38% of GPs and 62% of hospital doctors.

*‘Wanting a career that suits my domestic circumstances’* became much more important to choosers of general practice in the recent cohorts (*‘a great deal’* of importance for 83% in 2015, up from 47% in 1999). It also increased in importance for choosers of hospital specialties, but by a lesser amount (27% in 2015, up from 17% in 1999). *‘Wanting a job with acceptable hours and working conditions’* retained a huge level of importance to choosers of general practice (84% in 1999, 84% in 2015), but was less important and declined somewhat among doctors who chose hospital practice (40% in 1999, 32% in 2015). ‘*Availability of career posts’*, which barely registered as a consideration in 1999 when it was first asked about, was important to over 42% of 2015 responders who chose general practice and to 24% of responders who chose hospital specialties. *‘Future financial prospects’* were more important to GPs (16% in 2015) than hospital doctors (8% in 2015). Other changes across the cohorts were similar for those who chose general practice and those who chose hospital practice; differences in 2015 between GPs and hospital doctors were small (Figure 1 and Appendix 2).

### Sufficiency of exposure to general practice

In 1999, 2005, and 2015, doctors were presented with the statement *‘My exposure to general practice has been insufficient for me to assess it as a career option.’* Over all 3 survey years combined, 34% agreed or strongly agreed with the statement, 12% neither agreed nor disagreed, and 55% disagreed or strongly disagreed. The percentage who agreed fell from 39% in 1999 to 28% in 2015 (Table 2). Of those who did not seek a career in general practice, the percentage who agreed fell from 49% in 1999 to 32% in 2015, In other words, trends over time show that doctors felt better informed in recent than in earlier years about careers in general practice. This pattern was found among both males and females (see Table 2 and footnotes for test results, and Appendix 3 for details of all five response categories).

**Table 2.** Percentages of responding doctors who agreed with the statement *‘My exposure to general practice has been insufficient for me to assess it as a career option’* presented 3 years after graduation^a^

**Responders’ career choice**	**Sex**	**Year of survey**			

		**1999**	**2005**	**2015**	**All years**
All specialties	Male and female	38.7	29.4	27.7	32.4
	Male	42.7	33.2	30.0	36.4
	Female	35.3	27.1	26.2	29.7

General practice	Male and female	15.6	10.3	18.4	14.8
	Male	15.8	9.1	19.7	15.5
	Female	15.5	10.7	17.8	14.5

Hospital practice	Male and female	48.6	37.1	32.0	40.1
	Male	50.1	38.5	33.8	42.3
	Female	47.0	36.2	30.7	38.3

a *Based on responses from 6791 doctors. Excludes 27 who specified ‘no opinion’ and 221 who did not answer the statement. Responders in each survey year: 1999 — 1078 male, 1267 female, total 2345; 2005 — 743 male, 1290 female, total 2033; 2015 — 691 male, 1059 female, total 1750. Appendix 2 shows the numbers and percentages in each response category. Statistical tests on the percentages strongly agreeing or agreeing: 1) male versus female, all years combined: all career choices χ^2^ (1 degree of freedom [df])* = *37.8,* P*<0.001; general practice choices χ^2^ (1 df)* = *0.3,* P = *0.60; hospital practice choices χ^2^ (1 df) = 7.0,* P = *0.008; 2) year of survey comparisons on each row of the table (χ^2^ (2 df) tests): all* P*<0.001 except male GPs* (P = *0.04) and female GPs* (P *= 0.01).*

### Quality of GP training compared with hospital training (2012 graduates in 2015)

In 2015 the graduates of 2012 were presented with the statement *‘Generally speaking, GP training in the UK is of a higher quality than training in the hospital specialties.’* In all, 32% of the responders answered *‘no opinion’*, 23% agreed or strongly agreed, 19% neither agreed nor disagreed, and 26% disagreed or strongly disagreed (Table 3). There was no significant difference in agreement between males and females. Doctors who chose general practice were more likely to agree with the statement (36%) than doctors who chose hospital practice (17%).

**Table 3.** Percentages of responding doctors who agreed and disagreed with the statement ‘*Generally speaking, GP training in the UK is of a higher quality than training in the hospital specialties*’: responses in 2015 of the UK medical graduates of 2012^a^

**Career choice**	**Sex**	**Strongly agree/agree**	**Neither agree nor disagree**	**Strongly disagree/disagree**	**No opinion**	**Total**
All	Male and female	22.6	18.6	26.4	32.4	100 (*N* = 2012)
	Male	20.6	18.0	26.4	35.0	100 (*N*= 798)
	Female	23.9	19.0	26.4	30.7	100 (*N*= 1214)

General practice	Male and female	35.5	25.4	21.5	17.6	100 (*N*= 563)
	Male	35.4	26.5	22.2	15.9	100 (*N*= 189)
	Female	35.6	24.9	21.1	18.4	100 (*N*= 374)

Hospital medicine	Male and female	16.7	15.1	29.3	38.9	100 (*N*= 1208)
	Male	15.2	14.4	28.5	41.9	100 (*N*= 513)
	Female	17.8	15.5	29.9	36.7	100 (*N*= 695)

a *Statistical tests: male versus female: all career choices χ^2^ (3 degrees of freedom [df])* = *5.3,* P = *0.15; general practice choices χ^2^ (3 df)* = *0.7,* P = *0.88; hospital practice choices χ^2^ (3 df)* = *3.8,* P = *0.29.*

## DISCUSSION

### Summary

The attractiveness of a career in general practice relative to hospital practice, at least for these groups of doctors, has declined substantially in recent years. In response to the statement that general practice is a more attractive career than hospital practice, 59% of doctors agreed in the 1999 survey, 77% in 2005, and only 36% in 2015. The study statements were phrased in relative terms: aiming to find out how doctors regarded the appeal of general practice compared with the appeal of hospital practice. Over the 16 years covered by this study, the attractiveness of general practice has fallen relative to that of hospital practice. This may not necessarily reflect a decline in attractiveness of general practice in absolute terms; rather, it may reflect a larger increase, over time, in the appeal of hospital practice. Of factors specified by a higher percentage of aspiring hospital doctors than aspiring GPs in these surveys, enthusiasm for the specialty and good experiences of it (which may, of course, often be closely related) stand out. It would be deeply undesirable, of course, to try to reduce these in any way, in respect of the hospital specialties, in attempts to recruit more GPs. Further, it does not necessarily follow that finding a career attractive means that responders want to (or feel able to) pursue it. These findings instead should be seen in the context that, for many years, the percentage of UK-qualified doctors who seek a career in general practice has been far smaller than that needed to meet requirements for primary health care.

A specialty that fits the doctors’ domestic circumstances well has been much more important for aspiring GPs than hospital specialists ever since these surveys began. This has become even more important to choosers of general practice in recent cohorts; GPs’ ability to manage their hours and working conditions retained their importance. Any policies to reduce GPs’ ability to manage their work, or that adversely affect their work–life balance, may well have detrimental effects on recruitment.

Overall, one-third of doctors thought that their exposure to general practice had been insufficient for them to assess it as a career option, but fewer doctors rated their exposure as insufficient in the latest survey (2015) compared with 1999. Male doctors were more inclined than females to rate their exposure as insufficient. More doctors who had chosen careers in hospital practice rated their exposure to general practice as insufficient than doctors who had chosen general practice. It is important to ensure that all doctors feel that they have had enough exposure to general practice to be able to assess it as a career option.

### Strengths and limitations

These results are from an ongoing, longitudinal, national study that provides a view of doctors’ opinions about the attractiveness of general practice as a career. It has been possible to track how views have changed between 1999 and 2015. Generally, there have been good response rates to the surveys and the numbers of responders are large. As with all surveys, however, non-responder bias is a possibility, and it is acknowledged that the lower response rates in the more recent surveys may be an additional source of bias, although there is no evidence to suggest that responders are different from non-responders in characteristics relevant to this study. For example, 42.8% of non-responders from the 2012 cohort were male, compared with 39.7% of responders (*P* = 0.03).

### Comparison with existing literature

The present study details a decrease in the ratings of the attractiveness of general practice between 2005 and 2015. Research in the UK over the 10-year period from 2000 onwards found that career preferences for general practice among successive cohorts remained static over the years, with only one-fifth of cohorts naming it as an unequivocal first choice of career.^3^ Research in Switzerland has also found that general practice is not attractive as a career to medical students.^11^

It was found that 34% of doctors reported inadequate exposure to general practice. In a recent study, a similar percentage of Scottish Foundation doctors (37%) reported receiving career advice on general practice (compared with 50% reporting receiving career advice on hospital specialty training).^12^ This Scottish study also found that the most influential factors when considering a career in general practice were: undergraduate GP placement, discussion with peers and specialty trainees, and foundation GP placement. Therefore, the very factors that may persuade doctors to follow a career in general practice are the same factors to which doctors report receiving inadequate exposure.

### Implications for research

Further work is needed to establish whether the observed decrease in the relative attractiveness of general practice translates into a decreased preference for general practice as a career among more recent graduates. The attractiveness of general practice to current medical graduates is undoubtedly affected by their beliefs about GPs’ work–life balance, and their exposure to general practice in their training. GP choosers highly value hours and working conditions. This is clearly a key area in which to motivate doctors to choose general practice. It is noted that, in the most recent survey in 2015, the availability of career posts as a factor in motivating career choices became more important both among doctors choosing general practice and doctors choosing hospital practice. Improving the attractiveness of general practice is paramount to persuading the current generation of doctors to choose general practice in the large numbers required to meet government targets, especially when the most important determinants of general practice are enthusiasm and compatibility with family life.^13^^,^^14^

Studies from the same cohorts have been published previously on why some doctors initially choose, but then do not pursue, careers in different specialties.^13^^,^^15^ Comparatively few doctors actually considered general practice seriously and then rejected it. Of those who do want a career in general practice, most follow it through. The level of agreement between specialty choice expressed at year 1 after qualification and career specialty destination 10 years later is higher for general practice than for any other specialty.^16^ This suggests that doctors who do choose general practice enjoy working in it.^17^ The shortfall in doctors seeking a career in general practice is not accounted for by doctors considering it and rejecting it. Too few consider it at all.

The factors that influence specialty choice are a complex mix including the doctor’s interests, passion for the specialty, fascination with the job content, likely job satisfaction, aptitudes, likely ability to be successful in a chosen specialty, opportunities, compatibility with domestic and social life, material and intellectual rewards, and personal aspirations about how best to contribute to the service of patients. In a straightforward, brief, factual survey of career intentions, some of these are difficult to capture. In-depth studies are needed of motivators and interests to investigate what more might be done to achieve a better match between percentages of doctors wanting a career in general practice and percentages needed.

## Appendix 1. Levels of agreement with the statement *‘General practice is more attractive than hospital practice for doctors at present’* presented 3 years after graduation (numbers and percentages of responding doctors)^a^

**Table:**

**Career choice**	**Sex**	**Response**	**Year of survey**									

			**1999**		**2005**		**2011**		**2015**		**All years**	
				
			***N***	**%**	***N***	**%**	***N***	**%**	***N***	**%**	***N***	**%**
General practice	Male and female	Strongly agree	309	43.2	300	50.8	302	32.4	93	16.7	1004	35.9
		Agree	327	45.7	247	41.8	456	49.0	191	34.4	1221	43.7
		Neither	62	8.7	31	5.2	143	15.4	123	22.1	359	12.9
		Disagree	17	2.4	11	1.9	27	2.9	117	21.0	172	6.2
		Strongly disagree	0	0.0	2	0.3	3	0.3	32	5.8	37	1.3

	Male	Strongly agree	111	46.3	57	44.5	99	35.7	34	18.2	301	36.2
		Agree	96	40.0	62	48.4	130	46.9	54	28.9	342	41.1
		Neither	26	10.8	7	5.5	38	13.7	47	25.1	118	14.2
		Disagree	7	2.9	2	1.6	8	2.9	38	20.3	55	6.6
		Strongly disagree	0	0.0	0	0.0	2	0.7	14	7.5	16	1.9

	Female	Strongly agree	198	41.7	243	52.5	203	31.0	59	16.0	703	35.8
		Agree	231	48.6	185	40.0	326	49.8	137	37.1	879	44.8
		Neither	36	7.6	24	5.2	105	16.1	76	20.6	241	12.3
		Disagree	10	2.1	9	1.9	19	2.9	79	21.4	117	6.0
		Strongly disagree	0	0.0	2	0.4	1	0.2	18	4.9	21	1.1

Hospital medicine	Male and female	Strongly agree	114	7.2	405	28.6	354	19.0	74	6.5	947	15.7
		Agree	596	37.6	582	41.1	683	36.6	257	22.4	2118	35.2
		Neither	347	21.9	197	13.9	369	19.8	205	17.9	1118	18.6
		Disagree	452	28.5	170	12.0	349	18.7	401	35.0	1372	22.8
		Strongly disagree	78	4.9	61	4.3	113	6.0	209	18.2	461	7.7

	Male	Strongly agree	53	6.5	184	30.9	159	20.9	37	7.6	433	16.3
		Agree	318	39.1	240	40.3	295	38.7	94	19.4	947	35.7
		Neither	181	22.2	76	12.8	145	19.0	76	15.7	478	18.0
		Disagree	223	27.4	70	11.8	115	15.1	174	35.9	582	21.9
		Strongly disagree	39	4.8	25	4.2	48	6.3	104	21.4	216	8.1

	Female	Strongly agree	61	7.9	221	27.0	195	17.6	37	5.6	514	15.3
		Agree	278	36.0	342	41.7	388	35.1	163	24.7	1171	34.9
		Neither	166	21.5	121	14.8	224	20.3	129	19.5	640	19.0
		Disagree	229	29.6	100	12.2	234	21.2	227	34.3	790	23.5
		Strongly disagree	39	5.0	36	4.4	65	5.9	105	15.9	245	7.3

a This table excludes responses from doctors whose first career choice was tied, or whose first choice was not general practice or hospital medicine.

## Appendix 2. Trends between 1999 and 2015 in factors affecting career choices, for doctors who chose general practice and hospital practice: percentages of responders in each survey cohort who stated that each factor had a great deal of influence on their career choice^a^

**Table:**

**Factor**	**Year of survey**						

	**Choice**	**1999**	**2005**	**2011**	**2015**	χ^2^	***P*-value**
Domestic circumstances	GP	47.0	55.5	77.1	82.6	257.3	<0.001
	Hospital	17.3	15.4	28.9	27.3	91.5	<0.001

Hours/working conditions	GP	84.4	82.8	86.9	84.4	0.8	0.364
	Hospital	39.5	29.6	35.1	31.5	12.3	<0.001

Eventual financial prospects	GP	17.9	34.4	15.6	15.5	11.4	0.001
	Hospital	10.6	11.6	7.4	7.5	16.5	<0.001

Career and promotion prospects	GP	26.5	20.2	–	16.7	17.3	<0.001
	Hospital	28.7	17.5	–	18.4	40.9	<0.001

Self-appraisal of own skills	GP	46.6	46.8	55.6	50.6	8.7	0.003
	Hospital	50.1	40.5	59.4	51.8	23.4	<0.001

Advice from others	GP	14.2	27.8	–	16.3	0.2	0.625
	Hospital	19.3	17.4	–	19.4	0.2	0.666

Student experience of subject	GP	18.8	32.1	29.1	36.6	40.6	<0.001
	Hospital	25.5	30.6	35.4	38.0	65.3	<0.001

Inclinations before medical school	GP	17.1	15.2	17.9	15.3	0.03	0.854
	Hospital	15.3	12.6	12.6	15.5	0.3	0.570

Experience of jobs so far	GP	42.3	64.6	39.9	38.1	14.3	<0.001
	Hospital	63.5	76.9	64.0	62.4	4.8	0.029

Enthusiasm/commitment	GP	48.8	62.6	65.3	67.0	52.8	<0.001
	Hospital	60.6	69.8	86.5	90.5	504.5	<0.001

Availability of training places	GP	34.2	–	8.0	32.0	37.8	<0.001
	Hospital	30.1	–	7.6	14.3	248.5	<0.001

Availability of career posts	GP	1.7	–	8.2	42.0	258.9	<0.001
	Hospital	1.7	–	8.6	24.1	181.2	<0.001

a Statistics shown are the χ^2^ test for linear trend across the cohorts, and the associated P-value (1 degree of freedom).

## Appendix 3. Levels of agreement with the statement *‘My exposure to general practice has been insufficient for me to assess it as a career option’* presented 3 years after graduation (numbers and percentages of responding doctors)^a^

**Table:**

			**Year of survey**							

			**1999**		**2005**		**2015**		**All years**	
			
**Career choice**	**Sex**	**Response**	***N***	**%**	***N***	**%**	***N***	**%**	***N***	**%**
General practice	Male and female	Strongly agree	13	1.8	13	2.2	19	3.4	45	2.4
		Agree	97	13.8	48	8.1	84	15.0	229	12.4
		Neither	100	14.2	78	13.2	61	10.9	239	12.9
		Disagree	376	53.3	312	52.9	303	54.2	991	53.5
		Strongly disagree	119	16.9	139	23.6	92	16.5	350	18.9

	Male	Strongly agree	6	2.6	3	2.3	6	3.2	15	2.7
		Agree	31	13.2	9	6.8	31	16.5	71	12.8
		Neither	33	14.1	21	15.9	18	9.6	72	13.0
		Disagree	117	50.0	65	49.2	104	55.3	286	51.6
		Strongly disagree	47	20.1	34	25.8	29	15.4	110	19.9

	Female	Strongly agree	7	1.5	10	2.2	13	3.5	30	2.3
		Agree	66	14.0	39	8.5	53	14.3	158	12.2
		Neither	67	14.2	57	12.4	43	11.6	167	12.8
		Disagree	259	55.0	247	53.9	199	53.6	705	54.2
		Strongly disagree	72	15.3	105	22.9	63	17.0	240	18.5

Hospital medicine	Male and female	Strongly agree	152	9.3	117	8.1	97	8.1	366	8.6
		Agree	645	39.3	419	29.0	284	23.8	1348	31.5
		Neither	215	13.1	173	12.0	98	8.2	486	11.4
		Disagree	552	33.7	582	40.3	522	43.8	1656	38.7
		Strongly disagree	76	4.6	152	10.5	190	16.0	418	9.8

	Male	Strongly agree	85	10.1	60	9.8	41	8.2	186	9.5
		Agree	338	40.0	175	28.6	129	25.6	642	32.8
		Neither	127	15.0	72	11.8	49	9.7	248	12.7
		Disagree	256	30.3	234	38.3	194	38.6	684	34.9
		Strongly disagree	38	4.5	70	11.5	90	17.9	198	10.1

	Female	Strongly agree	67	8.4	57	6.9	56	8.1	180	7.8
		Agree	307	38.6	244	29.3	155	22.5	706	30.5
		Neither	88	11.1	101	12.1	49	7.1	238	10.3
		Disagree	296	37.2	348	41.8	328	47.7	972	42.0
		Strongly disagree	38	4.8	82	9.9	100	14.5	220	9.5

a This table excludes responses from doctors whose first career choice was tied, or whose first choice was not general practice or hospital medicine.
